# Factors Related to Life Satisfaction of Older Adults at Home: A Focus on Residential Conditions

**DOI:** 10.3390/healthcare10071279

**Published:** 2022-07-10

**Authors:** Jeong-Hye Park, Se-Won Kang

**Affiliations:** 1 Department of Nursing, Gyeongsang National University, Dongjin-ro 33, Jinju-si 52725, Korea; masternur@gnu.ac.kr; 2 Department of Nursing, Dongseo University, 47 Jurye-ro, Sasang-gu, Busan 47011, Korea

**Keywords:** older adults, life satisfaction, residential conditions, welfare policy

## Abstract

This study examined which residential conditions increase older adults’ life satisfaction at home. We used data from 8903 participants over 65 years old who did not need help in their daily lives from a 2020 survey of older adults conducted by the Korea Institute for Health and Social Affairs. Data analysis was conducted using descriptive statistics, independent sample *t*-test, Pearson correlation analysis, and hierarchical multiple regression with weights. The final model explained 34.2% of life satisfaction in old age. Residential conditions that increased life satisfaction the most in old age were the community environment (β = 0.16, *p* < 0.001) and satisfaction with the house (β = 0.15, *p* < 0.001). Other conditions that significantly affected life satisfaction were safety (β = 0.08, *p* < 0.001), interaction with neighbors (β = 0.08, *p* < 0.001), distance from children or relatives (β = 0.08, *p* < 0.001), frequency and route of public transportation (β = 0.05, *p* < 0.001), and abundance and distance of green spaces (β = 0.02, *p* = 0.031). Housing welfare policies should consider these results to increase life satisfaction for an increasingly aging population. Moreover, these data can be used to design age-friendly community environments.

## 1. Introduction

Korea is expected to become a super-aged society due to rapid aging. In fact, 16.5% of the country’s population was 65 years and older in 2021, and this figure is anticipated to grow to 20.3% by 2025 and to 43.9% by 2060 [1]. The median age in Korea is also higher than mid-40s; hence, the aging population is expected to grow faster than in any other country [2]. With increasing interest in the growing elderly population and their various welfare needs, stakeholders are also focusing on elderly’s life satisfaction. Further, some are emphasizing the importance of life quality during old age, as improving life expectancy has led to an increase in the number of elderly people [3].

Life satisfaction is the subjective evaluation of one’s life as a whole. It refers to the degree to which one feels that life is rich, meaningful, or valued compared to the standard of life being lived [4,5]. The higher the life satisfaction, the better the individual adjusts to it [6]. The evaluation of life satisfaction is generally based on one’s current life. Meanwhile, life satisfaction in old age can be used to evaluate whether one’s life has been successful overall. It can also be an indicator of adaptation to various changes and the reality of ageing [7]. Therefore, life satisfaction in old age may be more important than in other age groups. The life satisfaction of older adults in Korea is not high, while suicide and poverty rates are high [8]. To overcome these problems, it is necessary to identify factors that can increase life satisfaction in old age.

Notably, as the perspective on life in old age has become more realistic, efforts to understand and analyze various aspects related to life satisfaction in the elderly have greatly increased. Studies have identified the following factors related to life satisfaction in old age: health status [9,10], economic status [11], leisure activities, social participation such as volunteering [12,13], social relationships [14,15], living environment [16,17], computer use [18], employment status [19], and demographic characteristics [20].

The relationship between life satisfaction and one’s residence in old age is an especially important factor. In a narrow sense, a residence refers to the house in which an individual lives. Broadly, it refers to the house and community where they live [21,22]. The community includes green areas, parks, convenience facilities, public transportation, neighbors, and medical and welfare facilities. Individuals live their lives primarily in their house and community [23]. In particular, overall physical functions tend to decline with age. Further, older adults’ social and economic activities are diminished after they or their spouse retire. Thus, their activities tend to be limited to the house and surrounding areas [24,25].

Therefore, one’s residence can have a more meaningful impact on life in old age than in other age groups. As people age, the importance of their residence increase [26]. Older adults want to live in familiar and secure spaces. They feel that a convenient and practical house is sufficient, particularly when the space is safe; they have friendships and memories there. and the neighbors are friendly [27]. This indicates the psychosocial meaning associated with a residence in older age. Further, an individual’s psychosocial response to their place of residence can affect their life satisfaction.

Essentially, a residence has not only the meaning of protection and comfort for the elderly but also the social meaning of forming social relationships through interaction with the neighboring environment. Proper living and living environments not only enhance the elderly’s psychological and social well-being but also enrich their lives and improve their health. Therefore, one may predict that the more satisfied the elderly are with their living environment, the higher their life satisfaction.

Regarding residence and life satisfaction of the elderly in Korea, residence satisfaction is regarded as an important factor that influences quality of life [8]. Elderly people living in an environment with less tension and more individual choices have shown higher life satisfaction than those who do not. In addition, a pleasant living environment improves life satisfaction in old age.

Despite the importance of residences in the lives of the elderly, housing-related issues have been relatively neglected in policy responses to the elderly in Korea. The situation of elderly households is poor. Further, as the number of elderly households increases due to the rapid population aging and the corresponding national burden increases due to the dissolution of families, the challenge of elderly housing could become a major social problem [3].

As such, at the national level, the country will need to support the end of life while maintaining human dignity during this last stage by stabilizing residential life.

Then, a comprehensive residential policy for the elderly is needed. In an aging society where the living conditions and needs of the elderly are changing and diversified, appropriate policy solutions should be based on a comprehensive evaluation of current and future life conditions as well as on the subjective satisfaction of the elderly.

This study focuses on the relationship between residence-related factors and life satisfaction in various life areas that constitute the elderly’s life world. We aimed to identify the conditions needed for older adults to live a more satisfactory life in their place of residence. Our results can be used as baseline data to formulate future housing-related welfare policies for older adults.

### Study Purpose

This study aimed to investigate whether satisfaction with residence conditions is related to life satisfaction of older adults at home. Our aims were as follows:

(1) To investigate life satisfaction and satisfaction with residential conditions;
(2) To investigate the relationship between life satisfaction and satisfaction with residential conditions;
(3) To investigate factors related to life satisfaction.

## 2. Materials and Methods

### 2.1. Study Design

As a secondary analysis study, this study is a descriptive investigation of the relationship between satisfaction with residence conditions as a factor of life satisfaction, targeting the elderly living at home.

### 2.2. Study Participants and Sampling

This study used part of the raw data from a 2020 survey of the living profiles of the elderly [28] conducted by the Korea Institute for Health and Social Affairs. This survey is conducted every three years to identify older adults’ current status and characteristics to inform welfare policies and improve the quality of life of the growing older adult population. Data were collected through one-on-one direct interviews conducted from 14 September to November 2020 using the Tablet–PC-Assisted Personal Interview (TAPI) method by 169 interviewers, who had received training, based on a questionnaire designed by the research team.

The sample design of the survey on the elderly was first stratified by 17 cities and provinces in Korea; 9 provinces, except for 8 special cities and metropolitan cities, were divided into eastern parts, counties, and towns and stratified again.

The appropriate sample size was calculated to be approximately 10,000. Further, a two-step colony extraction method was used to prevent undersampling in areas with small populations using the square root of the number of elderly in the 2018 Population and Housing Census data. The final number of subjects in the raw data was 10,097; among these, 8903 subjects were selected for this study according to the subject selection criteria. The criteria were as follows: (1) elderly people who participated in the survey themselves, not their sons and daughters, or other people; (2) elderly people who were fully independent in all activities of daily living (ADL) and instrumental activities of daily living (IADL), that is, those who did not need assistance in their daily lives.

### 2.3. Data Collection

The survey on the status of the elderly is a legal survey based on the Elderly Welfare Act and is conducted regularly by the Korea Institute for Health and Social Affairs. 

In the 2020 Living Profiles of Older People Survey, the final version was completed by conducting a preliminary survey of 67 elderly people during the survey design process to collect data that represented the elderly population. Investigators were trained to conduct accurate interviews, and various inspections, such as field inspections and delivery of questionnaire guidelines, were conducted to ensure standardized and accurate investigations.

In addition, to control for survey quality, post-verification was conducted both during and after the survey. After data collection, the input was sent to a specialized service agency and computerized, and additional data were checked to minimize input errors.

Here, we conducted secondary data analysis using these raw data. To acquire data according to research progress, data use was approved on the Microdata Integration Service website [29].

### 2.4. Measurements

#### 2.4.1. Life Satisfaction 

Life satisfaction was measured on a 5-point Likert scale ranging from 1 (not satisfied at all) to 5 (very satisfied) for a single question: “How satisfied are you with your life as a whole these days?”. This item is the same as the life satisfaction item from the World Values Survey. Research has found similar results for single- and multi-item measures of life satisfaction [28]. A single item-based measure used in a basic study with questions considering age could also obtain accurate results [30].

#### 2.4.2. Satisfaction with Residence Condition

Evaluation of residence condition was based on eight items. The categories were “overall satisfaction with the housing you currently live in” [hereafter, “House”], including one item, and “satisfaction with the local community” including seven items. The latter included distance from convenience facilities (shops, social welfare facilities, medical institutions, etc.) [hereafter, “Facilities”]; satisfaction with the frequency and route of public transportation [hereafter, “Transportation”]; satisfaction with the abundance and distance of green spaces [hereafter, “Green space”]; satisfaction with security and transportation safety [hereafter, “Safety”]; satisfaction with interaction with neighbors [hereafter, “Neighbors”]; satisfaction with the distance from children or relatives [hereafter, “Distance”]; and satisfaction with the community environment [hereafter, “Environment”]. Each item was rated on a 5-point Likert scale ranging from 1 (not satisfied at all) to 5 (very satisfied).

#### 2.4.3. Characteristics of the Participants

Participants’ general characteristics included age, gender, education level, religion, marital status, residential area, type of housing, transportation, and family living together.

Health-related variables included perceived health status, number of chronic diseases, number of medications, and whether the participants had visited a hospital in the last month. The perceived health status question was “how is your health in general?”. It was rated on a 5-point Likert scale ranging from 1 (very bad) to 5 (very healthy).

Economic status-related variables included housing ownership, annual income, annual household income, real estate assets, financial assets, and debt.

### 2.5. Ethical Considerations

The data used here are public data accessed from the Microdata Integration Service homepage. These data were analyzed after approval [IRB No:2020-36] of the Ethics Committee of the Korea Institute for Health and Social Affairs. It was impossible to identify the participants because there was no personal identification information.

### 2.6. Data Analysis

Data were analyzed using the IBM SPSS Statistics version 23.0 program (IBM, Armonk, NY, USA).

(1) Participants’ characteristics, scores of life satisfaction, and satisfaction with residence condition were analyzed using frequency, percentage, mean, and standard deviation. Differences in life satisfaction according to satisfaction with residence condition were analyzed using an independent sample *t*-test.
(2) The relationship between life satisfaction and satisfaction with residential conditions was analyzed using the Pearson correlation coefficient.
(3) Hierarchical multiple regression analysis was performed with weights to identify factors with high explanatory power related to life satisfaction of older adults at home. Non-continuous variables were treated as dummy variables before analysis, and the suitability of the regression model was verified.
(4) The significance level was set at 0.05.

## 3. Results

### 3.1. Participants’ Characteristics 

The participants’ characteristics are shown in Table 1. The average age was 72.9 (±6.20) years, and 60.6% (*n* = 5394) were female. In addition, for 42.1% of the participants, education level was lower than elementary, 58.5% of them were religious, and 60.4% were married. More residents lived in provincial cities (46.3%) than in metropolitan cities, and the most common type of housing was apartment (47.4%). Participants’ main means of transportation was public transportation (71.8%), and a two-person household (i.e., living with a spouse) was the most common residence (52.2%).

Participants’ perceived health status scored 3.4 (±0.80) out of 5. We measured an average score of 1.7 (±1.33) for chronic diseases and of 1.6 (±1.39) for the use of different medications. We also found that 66.9% of the participants had visited the hospital in the last month; 80.8% of the participants owned their own house. The participants had an average annual income of KRW 1548.30 (±2431.78) million, a household income of KRW 2747.7 (±4075.80) million, real estate assets of KRW 23,017.2 (±33,889.66) million, financial assets of 3KRW 013.0 (±5068.49) million, and debt of KRW 1760.8 (±5487.93) million.

### 3.2. Life Satisfaction and Satisfaction with Residential Condition 

The average score for participant’s life satisfaction was 3.6 (±0.63). Residential condition satisfaction was as follows: “House”, 76.5%, “Facilities”, 70.2%, “Transportation”, 67.7%, “Green space”, 65.6%, “Safety”, 65.0%, “Neighbor”, 69.1%, “Distance”, 56.1%, and “Environment”, 65.0% (Table 2).

Further, life satisfaction differed according to satisfaction with residential conditions. The item with the largest difference was “Environment” (t = 34.61, *p* < 0.001), followed by “House” (t = 29.04, *p* < 0.001) (Table 2).

### 3.3. Relationship between Life Satisfaction and Satisfaction with Residential Conditions

Table 3 presents the Pearson correlation coefficients of the relationship between life satisfaction and various items related to satisfaction with residential conditions. Life satisfaction showed a significant correlation with all the residential conditions items. The item with the greatest correlation was community environment (r = 0.37, *p* < 0.001), followed by satisfaction with the house in which the participants lived (r = 0.35, *p* < 0.001).

### 3.4. Factors Related to Life Satisfaction

Hierarchical multiple regression analysis was performed to identify factors that increased life satisfaction in old age. The participants’ general characteristics, health status, economic status, and residence condition variables were captured sequentially. The suitability of the regression model was verified prior to the analysis. We found that the final regression equation was useful for explaining the dependent variable (F = 144.12, *p* < 0.001). The Durbin–Watson value was 1.90, tolerance was 0.22–0.99, and the variance expansion factor was 1.00–6.56, indicating autocorrelation and collinearity. There were no complications, and the regression model was suitable.

In the first stage when we considered general characteristics, the explanatory power of the independent variable with respect to the dependent variables was 10.2%. In the second stage, the explanatory power increased to 17.4% with the addition of the health status variable. When the economic state variable was added in the third stage, the explanatory power increased to 19.5%. Finally, the explanatory power was 34.2% in the final stage after adding the residence condition variables (Table 4).

The residence condition that increased life satisfaction the most was the community environment (β = 0.16, *p* < 0.001), followed by satisfaction with the house in which the participants lived (β = 0.15, *p* < 0.001). Other factors that improved life satisfaction were safety (β = 0.08, *p* < 0.001), interaction with neighbors (β = 0.08, *p* < 0.001), distance from children or relatives (β = 0.08, *p* < 0.001), satisfaction with public transportation and routes (β = 0.05, *p* < 0.001), and abundance and distance from green spaces (β = 0.02, *p* = 0.031). However, distance from convenient facilities was not a significant factor (β = −0.01, *p* = 0.425).

Other participants’ characteristics that were significantly related to an increase in life satisfaction were perceiving health to be good (β = 0.19, *p* < 0.001); owning a house (β = 0.08, *p* < 0.001); having higher annual income (β = 0.03, *p* = 0.019), real estate assets (β = 0.03, *p* = 0.013), and financial assets (β = 0.03, *p* = 0.002); lower age (β = −0.05, *p* < 0.001); more education (β = 0.07, *p* < 0.001); living in a rural area (β = 0.13, *p* < 0.001); being religious (β = 0.04, *p* = 0.002); living in a house (β = 0.02, *p* = 0.011); and primarily using private transportation (β = 0.04, *p* < 0.001) when respondents did not live with children or other people. This was the case for participants living in couple households (β = −0.03, *p* = 0.041; β = −0.04, *p* < 0.001).

## 4. Discussion

This study attempted to identify the residential conditions that lead to greater life satisfaction for older adults. This is important considering the rapid aging of the Korean population. We first investigated participants’ characteristics, including general, health-related, and economic characteristics. Second, we examined the variation in life satisfaction and in satisfaction with various residential conditions. Finally, we used hierarchical regression analysis to explore how general characteristics, health status, economic status, and residential condition affected life satisfaction.

Expectedly, the results show the importance of a residence and its various characteristics for increasing elders’ life satisfaction. For example, in the final regression model, the explanatory power of satisfaction with residence conditions for life satisfaction in old age was approximately twice that of health status and seven times higher than that of economic status. Overall, this result is consistent with the literature [8,25]. Older adults have indicated that a place of residence should be a home for simple living and that a convenient living space is sufficient. Further, psychological satisfaction affects life satisfaction in old age more than physical and economic factors do. Living in a community with favorable conditions for older adults can increase life satisfaction.

We found that satisfaction with the home and community environment increased elders’ life satisfaction the most; their effects were at least double that of other residential conditions.

A stable residence is essential for a comfortable life in old age. Older adults prioritize safety when moving to a new place of residence. When older adults who prioritize stability consider moving, it is primarily because of economically unavoidable or uncontrollable situations. They may be living in an old house, not having their own home, or living in a living space that is uncomfortable. Such involuntary movements can reduce life satisfaction. Indeed, we found that life satisfaction increased significantly as income and wealth increased when elders were living in their own house, in line with research [11]. Thus, satisfaction with the place in which one lives can be related to economic factors. This is the basic condition for increasing life satisfaction in old age and can be the focus of housing policies for older adults.

Community environment was another residence condition that increased life satisfaction in old age the most, in line with the literature [16,17,31]. Older adults’ community environment is often kept sanitary and clean so that people can enjoy a healthy and comfortable environment. Benches or public toilets, wide gentle surfaces, pedestrian paths with high boundary stones, accessible locations, well-marked crossings, and ample green spaces contribute to life satisfaction. Furthermore, sufficient green space also increases life satisfaction in old age because it produces clean air and positively influences subjective health [32,33,34]. Subjective health is another significant factor [35,36] that we identified as influencing life satisfaction. Therefore, it is necessary to improve the community environment in an age-conscious manner while considering these factors. An age-friendly environment can improve the quality of life of the elderly and reduce intergenerational conflict. It can also increase trust and solidarity within the community, implying that age-friendly community environments benefit everyone.

Notably, the findings show that satisfaction with residential convenience facilities was not a significant factor related to life satisfaction in older age. Satisfaction with public transportation had little effect on life satisfaction. Rather, residence safety, interaction with neighbors, and distance from children or relatives had a more significant influence on life satisfaction.

This study has several limitations. First, because secondary data were used, its scope was limited to variables on which data were collected. Future studies should supplement the qualitative research data used here with direct research and participant interviews. Second, this study used a cross-sectional survey; hence, we cannot make any assertions about causal relationships. Future studies should consider extending the scope using a cross-sectional design. Nevertheless, this study is meaningful in that it used large-scale national survey data to demonstrate that living conditions in old age have a great influence on life satisfaction.

## 5. Conclusions

This study demonstrated that residential conditions are an essential factor for increasing the life satisfaction of older adults living in local communities. These residential conditions include stable living situations, proximity to amenities and conveniences, and a clean and comfortable age-friendly community environment.

These conditions indicate that elders’ residences should be havens for a comfortable and secure life. Importantly, policymakers should consider these factors when drafting housing-related welfare policies for the elders to improve the life satisfaction of an increasingly aging population.

## Figures and Tables

**Table 1:** Participant characteristics (*n* = 8903).

Variables			N (%) or Mean ± SD
Age (years)			72.9 ± 6.20
Gender	Male		3509 (39.4)
	Female		5394 (60.6)
Educational level	≤Elementary school		3750 (42.1)
	Middle school		2170 (24.4)
	High school		2519 (28.3)
	≥College		463 (5.2)
Religion	Yes		5211 (58.5)
	No		3692 (41.5)
Marital status	Married		5379 (60.4)
	Single		34 (0.4)
	Divorced		310 (3.5)
	Widowed		3128 (35.1)
	Separated		52 (0.6)
Residential area	Seoul/satellite cities		1995 (22.4)
	Metropolitan cities		2788 (31.3)
	Provincial cities		4120 (46.3)
Type of housing	Detached home		3506 (39.4)
	Apartment		4218 (47.4)
	Multi-unit house		1100 (12.4)
	Others		79 (0.9)
Transportation	Public transportation		6394 (71.8)
	Owner-driven car		2217 (24.9)
	Others		292 (3.3)
Family living together	Living alone		2736 (30.7)
	Living with spouse only		4644 (52.2)
	Living with child		1405 (15.8)
	Others		118 (1.3)
Health-related	Perceived health status		3.4 ± 0.80
	Number of chronic diseases		1.7 ± 1.33
	Number of medications		1.6 ± 1.39
	Visiting hospital last month		5959 (66.9)
Economic status-related	Housing ownership	Own one’s house	7191 (80.8)
		On lease	1433 (16.1)
		Free housing	279 (3.1)
	Annual income *		1548.3 ± 2431.78
	Annual household income *		2747.7 ± 4075.80
	Real estate assets *		23,017.2 ± 33,889.66
	Financial assets *		3013.0 ± 5068.49
	Debt *		1760.8 ± 5487.93

* Korean won, tens of thousands, SD: Standard deviation.

**Table 2:** Life satisfaction and satisfaction with residential condition (*n* = 8903).

Variables		*n* (%)	Life Satisfaction Score (Range: 1–5) (Mean ± SD)	t(*p*)
Life satisfaction total score		-	3.6 ± 0.63	-
Residential condition satisfaction with *:				
House	Satisfied	6813 (76.5)	3.6 ± 0.63	29.04 (<0.001)
	Not satisfied	2090 (23.5)	3.2 ± 0.67	
Facilities	Satisfied	6247 (70.2)	3.6 ± 0.66	18.42 (<0.001)
	Not satisfied	2656 (29.8)	3.3 ± 0.67	
Transportation	Satisfied	6024 (67.7)	3.6 ± 0.66	15.89 (<0.001)
	Not satisfied	2879 (32.3)	3.4 ± 0.67	
Green space	Satisfied	5838 (65.6)	3.6 ± 0.66	22.19 (<0.001)
	Not satisfied	3065 (34.4)	3.3 ± 0.65	
Safety	Satisfied	5789 (65.0)	3.6 ± 0.65	22.69 (<0.001)
	Not satisfied	3114 (35.0)	3.3 ± 0.66	
Neighbor	Satisfied	6153 (69.1)	3.7 ± 0.65	26.08 (<0.001)
	Not satisfied	2750 (30.9)	3.3 ± 0.64	
Distance	Satisfied	4991 (56.1)	3.7 ± 0.65	24.83 (<0.001)
	Not satisfied	3912 (43.9)	3.3 ± 0.65	
Environment	Satisfied	5783 (65.0)	3.7 ± 0.62	34.61 (<0.001)
	Not satisfied	3120 (35.0)	3.2 ± 0.65	

* House—satisfaction with the housing you currently live in; Facilities—satisfaction with the local community including distance from convenience facilities; Transportation—satisfaction with frequency and route of public transportation; Green space—satisfaction with abundance and distance of green spaces; Safety—satisfaction with security and transportation safety; Neighbors—satisfaction with interaction with neighbors; Distance—satisfaction with distance from children or relatives; Environment—satisfaction with the community environment.

**Table 3:** Relationship between life satisfaction and satisfaction with residential conditions.

Variables	A	B	C	D	E	A	G	H	I
Life satisfaction (A)	1								
Residential condition satisfaction with									
House (B)	0.35 (<0.001)	1							
Facilities (C)	0.24 (<0.001)	0.19 (<0.001)	1						
Transportation (D)	0.24 (<0.001)	0.18 (<0.001)	0.60 (<0.001)	1					
Green space (E)	0.28 (<0.001)	0.25 (<0.001)	0.31 (<0.001)	0.32 (<0.001)	1				
Safety (F)	0.29 (<0.001)	0.19 (<0.001)	0.73 (<0.001)	0.43 (<0.001)	0.46 (<0.001)	1			
Neighbor (G)	0.32 (<0.001)	0.22 (<0.001)	0.29 (<0.001)	0.33 (<0.001)	0.40 (<0.001)	0.37 (<0.001)	1		
Distance (H)	0.31 (<0.001)	0.22 (<0.001)	0.41 (<0.001)	0.38 (<0.001)	0.37 (<0.001)	0.40 (<0.001)	0.41 (<0.001)	1	
Environment (I)	0.37 (<0.001)	0.25 (<0.001)	0.49 (<0.001)	0.46 (<0.001)	0.48 (<0.001)	0.52 (<0.001)	0.56 (<0.001)	0.45 (<0.001)	1

A: Life satisfaction, B: House—satisfaction with the housing you currently live in, C: Facilities—satisfaction with the local community including distance from convenience facilities, D: Transportation—satisfaction with frequency and route of public transportation, E: Green space—satisfaction with abundance and distance of green space, F: Safety—satisfaction with security and transportation safety, G: Neighbors—satisfaction with interaction with neighbors, H: Distance—satisfaction with distance from children or relatives, I: Environment—satisfaction with the community environment.

**Table 4:** Factors related to life satisfaction.

Variables		Step I			Step II			Step III			Step IV		
		β	SE	*p*	β	SE	*p*	β	SE	*p*	β	SE	*p*
Age		−0.11	0	<0.001	−0.04	0	0.001	−0.04	0	0.001	−0.05	0	<0.001
Gender (ref. = male)		−0.01	0.10	0.227	−0.01	0.01	0.237	−0.01	0.01	0.185	−0.01	0.01	0.565
Education level (ref. = ≤elementary)	Middle	0.12	0.02	<0.001	0.08	0.02	<0.001	0.07	0.02	<0.001	0.05	0.01	<0.001
	≥High	0.17	0.03	<0.001	0.12	0.03	<0.001	0.10	0.03	<0.001	0.07	0.03	<0.001
Religion (ref. = no)		0.03	0.01	0.002	0.05	0.01	<0.001	0.05	0.01	<0.001	0.04	0.01	<0.001
Marital status: spouse (ref. = no)		0.01	0.03	0.636	0.01	0.03	0.745	−0.01	0.03	0.536	0	0.03	0.808
Residential area (ref. = Seoul/satellite cities)	Metropolitan cities	0.06	0.02	<0.001	0.06	0.02	<0.001	0.06	0.02	<0.001	0.05	0.02	<0.001
	Provincial cities	0.13	0.02	<0.001	0.14	0.02	<0.001	0.14	0.02	<0.001	0.13	0.02	<0.001
Type of housing (ref. = Apartment)		−0.04	0.01	<0.001	−0.05	0.01	<0.001	−0.03	0.01	0.002	0.02	0.01	0.011
Transportation (ref. = public)	Owner-driven car	0.10	0.02	<0.001	0.08	0.02	<0.001	0.05	0.02	<0.001	0.04	0.02	<0.001
	Others	−0.04	0.04	<0.001	−0.04	0.04	<0.001	−0.05	0.04	<0.001	−0.04	0.03	<0.001
Family living together (ref. = alone)	With spouse only	0.02	0.04	0.371	0.01	0.03	0.773	−0.00	0.03	0.901	−0.01	0.03	0.586
	With child	0.01	0.03	0.406	0.01	0.03	0.344	−0.01	0.03	0.707	−0.03	0.02	0.041
	Others	−0.04	0.06	<0.001	−0.03	0.06	0.002	−0.03	0.06	0.003	−0.04	0.05	<0.001
Health-related	Perceived health status				0.28	0.01	<0.001	0.27	0.01	<0.001	0.19	0.01	<0.001
	Number of Chronic diseases				0.00	0.01	0.894	−0.02	0.01	0.429	−0.01	0.01	0.503
	Number of medication				−0.03	0.01	0.153	−0.01	0.01	0.488	−0.03	0.01	0.065
	Visiting hospital (ref. = no)				−0.01	0.02	0.381	−0.02	0.02	0.152	−0.01	0.01	0.317
Economic status-related	Own house (ref. = others)							0.10	0.02	<0.001	0.08	0.02	<0.001
	Annual income							0.04	0	0.005	0.03	0	0.019
	Annual household income							0.02	0	0.292	0.01	0	0.437
	Real estate assets							0.05	0	<0.001	0.03	0	0.013
	Financial assets							0.04	0	0.001	0.03	0	0.002
	Debt							0	0	0.834	0	0	0.881
Residential condition satisfaction	House										0.15	0.01	<0.001
	Facilities										−0.01	0.01	0.425
	Transportation										0.05	0.01	<0.001
	Green space										0.02	0.01	0.031
	Safety										0.08	0.01	<0.001
	Neighbor										0.08	0.01	<0.001
	Distance										0.08	0.01	<0.001
	Environment										0.16	0.01	<0.001
R^2^		0.102			0.174			0.195			0.342		
Adjusted R^2^		0.101			0.172			0.193			0.340		
R^2^ Change		-			0.072			0.021			0.147		
F(*p*)		72.10 (<0.001)			103.89 (<0.001)			89.57 (<0.001)			144.12 (<0.001)		

## Data Availability

Data sharing is not applicable; no new data were created or analyzed in this study.

## References

[1] Korean Statistical Information Service Population Structure by Age. http://www.index.go.kr/potal/main/EachDtlPageDetail.do?idx_cd=1010#quick_02.

[2] Korean Statistical Information Service 2021 Senior Citizen Statistics. https://kostat.go.kr/portal/korea/kor_nw/1/1/index.board?bmode=read&aSeq=403253.

[3] Korean Institute for Health and Social Affairs 2020 Survey of the Elderly. https://mdis.kostat.go.kr/index.do.

[4] Celik S.S., Celik Y., Hikmet N., Khan M.M. (2018). Factors affecting life satisfaction of older adults in Turkey. Int. J. Aging Hum. Dev..

[5] Van Damme-Ostapowicz K., Cybulski M., Galczyk M., Krajewska-Kulak E., Sobolewski M., Zalewska A. (2021). Life satisfaction and depressive symptoms of mentally active older adults in Poland: A cross-sectional study. BMC Geriatr..

[6] Sharif S.P., Amiri M., Allen K.-A., Nia H.S., Fomani F.K., Matbue Y.H., Goudarzian A.H., Arefi S., Yaghoobzadeh A., Waheed H. (2021). Attachment: The mediating role of hope, religiosity, and life satisfaction in older adults. Health Qual. Life Outcomes.

[7] Tian H.M., Wang P. (2021). The role of perceived social support and depressive symptoms in the relationship between forgiveness and life satisfaction among older people. Aging Ment. Health.

[8] Kim K.Y., Park H.B. (2020). Factors influencing senior citizens’ life satisfaction. Korean Public Manag. Rev..

[9] Rodgers V., Neville S., La Grow S. (2017). Health, functional ability and life satisfaction among older people 65 years and over: A cross-sectional study. Contemp. Nurse.

[10] Phulkerd S., Thapsuwan S., Chamratrithirong A., Gray R.S. (2021). Influence of healthy lifestyle behaviors on life satisfaction in the aging population of Thailand: A national population-based survey. BMC Public Health.

[11] Kim B.J., Chen L., Xu L., Lee Y. (2021). Self-Rated health and subjective economic status in life satisfaction among older Chinese immigrants: A cross-sectional study. Healthcare.

[12] Yoon H., Lee W.S., Kim K.B., Moon J. (2020). Effects of leisure participation on life satisfaction in older Korean adults: A panel analysis. Int. J. Environ. Res. Public Health.

[13] Jongenelis M.I., Jackson B., Newton R.U., Pettigrew S. (2022). Longitudinal associations between formal volunteering and well-being among retired older people: Follow-up results from a randomized controlled trial. Aging Ment. Health.

[14] Zhou J. (2018). Improving older people’s life satisfaction via social networking site use: Evidence from China. Australas. J. Ageing.

[15] Shimkhada R., Tse H.W., Ponce N.A. (2022). Life satisfaction and social and emotional support among Asian American older adults. J. Am. Board Fam. Med..

[16] Zhou K., Tan J., Watanabe K. (2021). How does perceived residential environment quality influence life satisfaction? Evidence from urban China. J. Community Psychol..

[17] Ng S.I., Lim X.J., Hsu H.C. (2021). The importance of age-friendly city on older people’s continuity and life satisfaction. Int. J. Environ. Res. Public Health.

[18] Yong J.C., Toh W.X., Lee S.T., Tng G.Y., Tov W. (2020). Cognitive, social, emotional, and subjective health benefits of computer use in adults: A 9-year longitudinal study from the Midlife in the United States (MIDUS). Comput. Hum. Behav..

[19] Boyce C.J., Brown G.D., Moore S.C. (2010). Money and happiness: Rank of income, not income, affects life satisfaction. Psychol. Sci..

[20] Liu B.C., Leung D.S., Warrener J. (2019). The Interaction effect of gender and residential environment, individual resources, and needs satisfaction on quality of life among older adults in the United Kingdom. Gerontol. Geriatr. Med..

[21] Amián J.G., Alarcón D., Fernández-Portero C., Sánchez-Medina J.A. (2021). Aging living at home: Residential satisfaction among active older adults based on the perceived home model. Int. J. Environ. Res. Public Health.

[22] Moor N.J.A., Hamers K., Mohammadi M. (2022). Ageing well in small villages: What keeps older adults happy? Environmental indicators of residential satisfaction in four Dutch villages. Int. J. Environ. Res. Public Health.

[23] Lin S., Huang Y. (2018). Community environmental satisfaction: Its forms and impact on migrants’ happiness in urban China. Health Qual. Life Outcomes.

[24] Yeung P., Severinsen C., Good G., O’Donoghue K. (2022). Social environment and quality of life among older people with diabetes and multiple chronic illnesses in New Zealand: Intermediary effects of psychosocial support and constraints. Disabil. Rehabil..

[25] Jolanki O.H. (2021). Senior housing as a living environment that supports well-being in old age. Front. Public Health.

[26] Lahti A.M., Mikkola T.M., Salonen M., Wasenius N., Sarvimäki A., Eriksson J.G., von Bonsdorff M.B. (2021). Mental, physical and social functioning in independently living senior house residents and community-dwelling older adults. Int. J. Environ. Res. Public Health.

[27] Zimmermann J., Hansen S., Wagner M. (2021). Home environment and frailty in very old adults. Z. Gerontol. Geriatr..

[28] Cheung F., Lucas R.E. (2014). Assessing the validity of single-item life satisfaction measures: Results from three large samples. Qual. Life Res..

[29] Microdata Integrated Service 2020 Survey of the Elderly. Korean Institute For Health and Social Affairs. https://www.kihasa.re.kr/en.

[30] Ji E.J., Kim J., Son D.K. (2016). Basic Study for Ageism Scale Development.

[31] Huang F., Fu P. (2021). Intergenerational support and subjective wellbeing among oldest-old in China: The moderating role of economic status. BMC Geriatr..

[32] Jeste D.V., Blazer D.G., Buckwalter K.C., Cassidy K.-L.K., Fishman L., Gwyther L.P., Levin S.M., Phillipson C., Rao R.R., Schmeding E. (2016). Age-friendly communities initiative: Public health approach to promoting successful aging. J. Geriatr. Psychiatry.

[33] Abraham C.S., Villeneuve P.J., Raina P., Griffith L.E., Rainham D., Dales R., Peters C.E., Ross N.A., Crouse D.L. (2022). Increased urban greenness associated with improved mental health among middle-aged and older adults of the Canadian Longitudinal Study on Aging (CLSA). Environ. Res..

[34] Crouse D.L., Pinault L., Christidis T., Lavigne E., Thomson E.M., Villeneuve P.J. (2021). Residential greenness and indicators of stress and mental well-being in a Canadian national-level survey. Environ. Res..

[35] Dumitrache C.G., Rubio L., Rubio-Herrera R. (2017). Perceived health status and life satisfaction in old age, and the moderating role of social support. Aging Ment. Health.

[36] Rapoport M. (2019). Life satisfaction in older adults in Spain: Beyond subjective health. Int. Psychogeriatr..

